# Supplementary material for: Inhibition of MACC1-Induced Metastasis in Esophageal and Gastric Adenocarcinomas
Source: Cancers (Basel). 2022 Mar 31;14(7):1773. doi: 10.3390/cancers14071773 (PMC8997092; doi:10.3390/cancers14071773)
Supplement: Supplementary file 1 [file cancers-14-01773-s001.zip › cancers-1614068-supplementary.pdf]

.Supplement

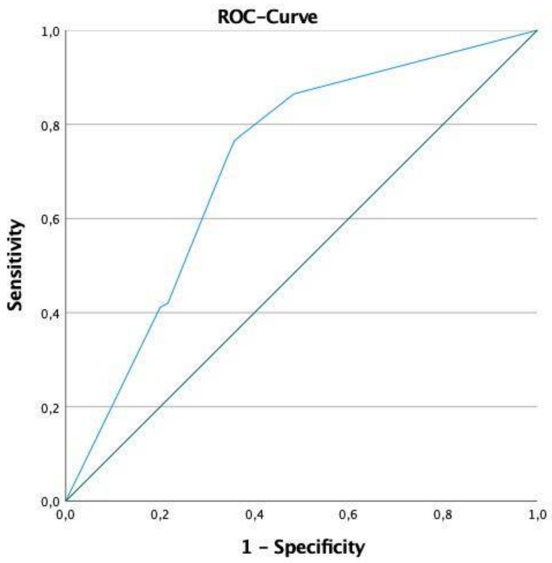

Figure S1. ROC Curve for IRS cut-off analysis (AUC 0.716 [0.656 – 0.776]).

Table S1. Sensitivity, specificity and Youden Index for different IRS cut-off values.

Positive if Greater than or Equal to	Sensitivity	1- Specificity	Specificity	Youden Index
−1	1	1	0	0
1	0.869	0.500	0.500	0.369
3	0.864	0.483	0.517	0.381
**5**	**0.766**	**0.358**	**0.642**	**0.408**
7	0.729	0.342	0.658	0.387
8,5	0.421	0.217	0,783	0.204
10,5	0.411	0.200	0.800	0.211
13	0	0	1	0

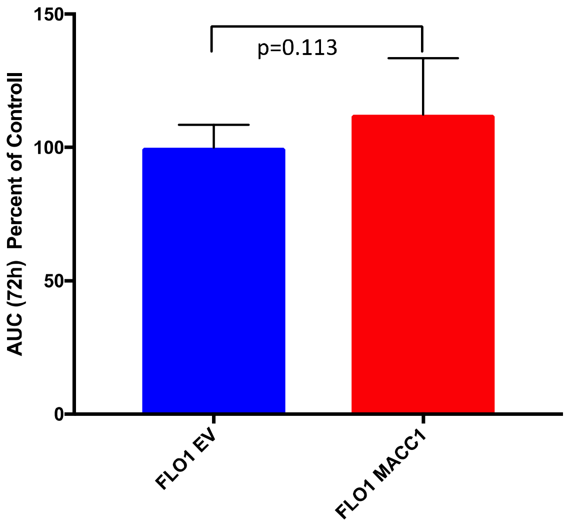

Figure S2. Proliferation of FLO1 EV and FLO1 MACC1 over 72 h. Analysis of in vitro proliferation assay.
